# Airborne Transmission of Melioidosis to Humans from Environmental Aerosols Contaminated with *B*. *pseudomallei*

**DOI:** 10.1371/journal.pntd.0003834

**Published:** 2015-06-10

**Authors:** Pei-Shih Chen, Yao-Shen Chen, Hsi-Hsun Lin, Pei-Ju Liu, Wei-Fan Ni, Pei-Tan Hsueh, Shih-Hsiung Liang, Chialin Chen, Ya-Lei Chen

**Affiliations:** 1 Department of Public Health, Kaohsiung Medical University, Kaohsiung, Taiwan; 2 Department of Internal Medicine, Kaohsiung Veterans General Hospital, Kaohsiung, Taiwan; 3 Department of Medicine, E-Da Hospital, Kaohsiung, Taiwan; 4 Department of Biotechnology, National Kaohsiung Normal University, Kaohsiung, Taiwan; 5 Department of Biological Sciences, National Sun Yat-Sen University, Kaohsiung, Taiwan; 6 Center of Research, Diagnostics and Vaccine Development, Centers for Disease Control (ROC), Taipei, Taiwan; University of Tennessee, UNITED STATES

## Abstract

Melioidosis results from an infection with the soil-borne pathogen *Burkholderia pseudomallei*, and cases of melioidosis usually cluster after rains or a typhoon. In an endemic area of Taiwan, *B*. *pseudomallei* is primarily geographically distributed in cropped fields in the northwest of this area, whereas melioidosis cases are distributed in a densely populated district in the southeast. We hypothesized that contaminated cropped fields generated aerosols contaminated with *B*. *pseudomallei*, which were carried by a northwesterly wind to the densely populated southeastern district. We collected soil and aerosol samples from a 72 km^2^ area of land, including the melioidosis-clustered area and its surroundings. Aerosols that contained *B*. *pseudomallei*-specific TTSS (type III secretion system) ORF2 DNA were well distributed in the endemic area but were rare in the surrounding areas during the rainy season. The concentration of this specific DNA in aerosols was positively correlated with the incidence of melioidosis and the appearance of a northwesterly wind. Moreover, the isolation rate in the superficial layers of the contaminated cropped field in the northwest was correlated with PCR positivity for aerosols collected from the southeast over a 2-year period. According to pulsed-field gel electrophoresis (PFGE) and multilocus sequence typing (MLST) analyses, PFGE Type Ia (ST58) was the predominant pattern linking the molecular association among soil, aerosol and human isolates. Thus, the airborne transmission of melioidosis moves from the contaminated soil to aerosols and/or to humans in this endemic area.

## Author Summary

Cases of melioidosis, an emerging disease, are usually clustered after rains or typhoons. In Taiwan, the endemic area includes rural cropped fields contaminated with *B*. *pseudomallei* in the northwest and a densely populated district in the southeast, where melioidosis-cluster is distributed. We hypothesized that contaminated cropped fields generated aerosols contaminated with *B*. *pseudomallei*, which were carried by a northwesterly wind and enveloped the southeastern densely populated district. In a prospective study, aerosols that contained *B*. *pseudomallei*-specific DNA were well distributed in the densely populated districts but were rarely found in their surrounding areas. The concentration of specific DNA in aerosols correlated with the melioidosis incidence and the northwesterly wind in this endemic area. The isolation rate in the superficial layers of the contaminated cropped field in the northwest correlated with the PCR positivity of aerosols collected from the southeastern district over a 2-year period. We used genetic typing to identify a molecular association among the soil, aerosols and human isolates. We suggest that melioidosis is airborne and is transmitted from the contaminated soils to aerosols and/or to humans in this area.

## Introduction

The emerging infectious disease melioidosis is caused by *Burkholderia pseudomallei*, which is commonly isolated from soil and water in endemic areas such as Southeast Asia and northern Australia [1–5]. Melioidosis cases usually cluster during the rainy season, particularly after the appearance of a heavy rainfall, cyclone or typhoon [6–9]. Flooding enriches the nutrients in the soil, and the pathogen consequently grows and moves from contaminated sites to remote areas *via* water [10–11]. Humans acquire melioidosis from directly contacting, ingesting or inhaling contaminated soil or dusts [12–14]. In humans, a melioidosis outbreak was previously linked to drinking water contaminated with *B*. *pseudomallei* [15]. Subcutaneous inoculation is recognized as a major transmission mode because farmers have an occupational risk of melioidosis [16]. Additionally, some melioidosis patients exhibit obvious skin wounds, and athletes can acquire melioidosis from contact with contaminated soil while playing sports [17–18]. Travelers can develop melioidosis (with pneumonia) *via* inhalation during a helicopter flight [12]. This aerosolization of dry dust associated with the melioidosis incidence was identified in helicopter crews after the Vietnam War [19]. According to epidemiological evidence, pulmonary melioidosis can be acquired by inhalation after heavy rainfalls and strong winds [6,11]. In animal experiments, mice that inhaled aerosols contaminated with *B*. *pseudomallei* developed severe inflammation and neutrophil infiltration in the lungs, which mimicked the symptoms of human pulmonary melioidosis [20].

We previously identified clusters of melioidosis cases in an endemic area (the Zoynan region) in Taiwan [21]. The melioidosis incidence in this endemic area is approximately 10–20 cases per 100,000 persons, which is similar to the annual incidence in Thailand (7.98–21.3 cases per 100,000 persons) and northern Australia (5.4–24.2 cases per 100,000 persons) [22–23]. The incidence of melioidosis (over 70% manifested with pulmonary melioidosis) was associated with the appearance of heavy rainfalls and strong winds such as typhoons [11]. Previously, we demonstrated that aerosols containing *B*. *pseudomallei*-specific DNA appeared during the typhoon season but not during the dry season (in which no typhoon occurred) [24]. This endemic area comprises hilly regions in the south and cropped fields in the north [21]. Clusters of melioidosis cases have appeared north of the hills, as far as the cropped fields (approximately 5 km away) [11]. Because a strong northwest wind originating from a counterclockwise cyclone occurred during the typhoon season, we hypothesized that the contaminated aerosols were carried by northwest winds from cropped fields contaminated with *B*. *pseudomallei* and enveloped the endemic area during the typhoon season. The number of melioidosis cases was associated with contaminated aerosol inhalation during typhoon seasons due to pathogen-carrying winds. To test this hypothesis, we integrated relevant data, which showed that *B*. *pseudomallei* was geographically distributed in northwestern cropped soil, that aerosols contaminated with *B*. *pseudomallei*-specific DNA were increased during the typhoon season, that the DNA concentration was related to the incidence of melioidosis and the appearance of a northwesterly wind and that aerosol isolates were genetically identical to human isolates.

## Methods

### Ethics statement

In this study, we adhered to the rules of the Personal Information Protection Act (Taiwan) by anonymizing the melioidosis patient data. Viable *B*. *pseudomallei* were manipulated in an airflow-controlled lab (BSL III level), and these procedures were approved by the Institutional Biosafety Committee (NKNU, Taiwan).

### Study areas and data collection

**Fig 1:**
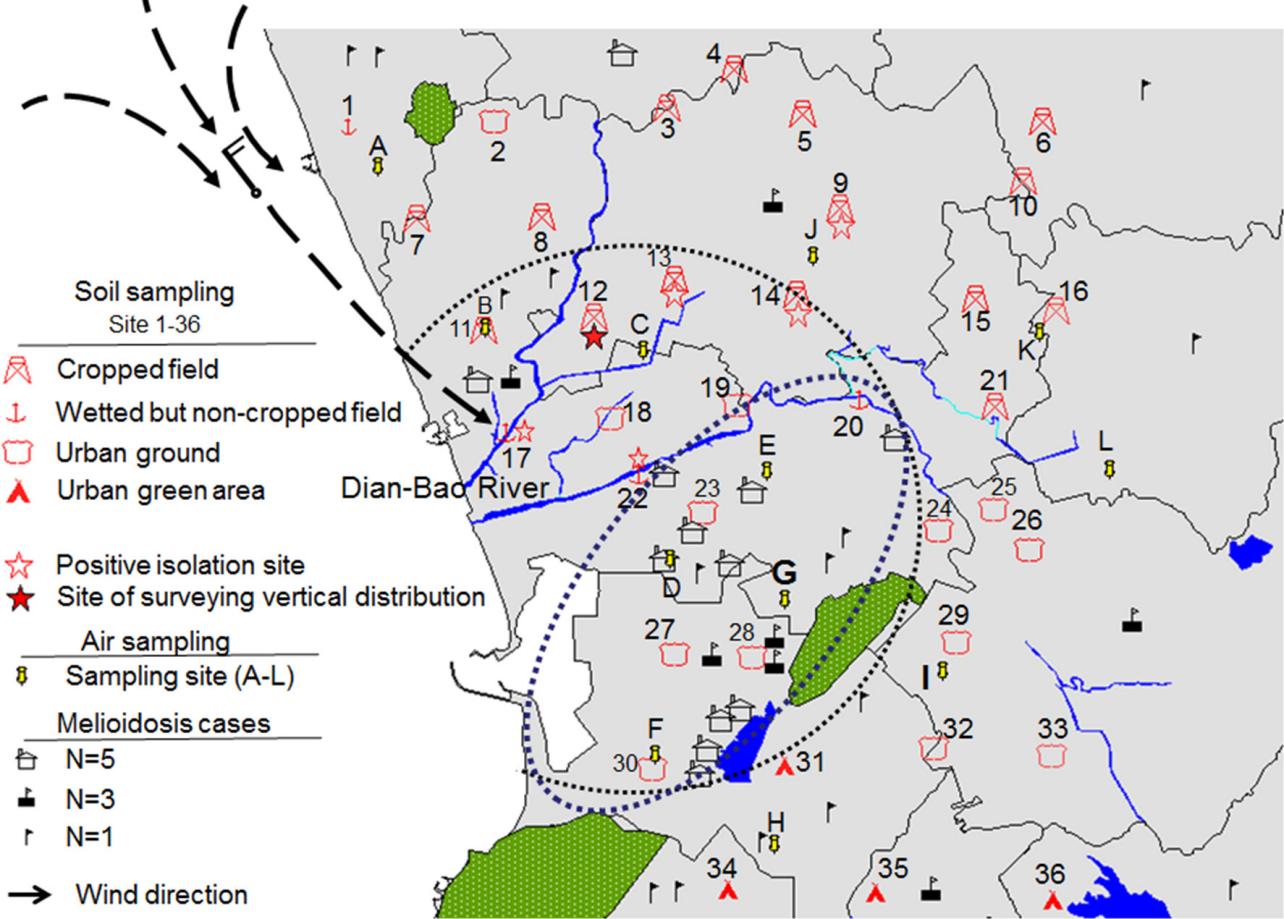
The distribution of melioidosis cases and sampling sites. Using a random sampling strategy, the locations of sampling sites of the cropped field, wetted but non-cropped field, urban ground or urban green area are shown (legends on left of the figure). The positive isolation sites of the sampling sites are labeled with hollow or solid red stars. The solid red star indicates a site that was sampled using the fixed-interval strategy. The melioidosis locations are indicated by different symbols representing the number of melioidosis cases (see left on the figure). The circle on the map indicates the endemic areas of melioidosis (n = 75, defined in a previous study [21]). An ellipse on the map indicates the core region of melioidosis cases (n = 68, defined by this study). The hypothesis is that aerosols contaminated with *B*. *pseudomallei* are carried by wind (wind direction indicated by dotted lined with a directional arrow) from the northwestern contaminated cropped field (red star) and envelop the endemic area, particularly the core region, during the rainy (typhoon) season.

The study areas were approximately 72 km^2^, including an endemic area and its surroundings (Fig 1). Using a Moran *I* analysis, which consisted of a spatial autocorrelation measurement, we previously observed that the melioidosis incidence was higher in the endemic area than in the neighboring areas [21]. As shown in Fig 1, the topographies of rivers, lakes and hills were mapped (MapInfo, v. 7.0) using GPS coordinates from Google Earth. The land north of the Dian-Bao River is utilized to grow rice, pineapple, sugarcane and vegetables, while a densely populated district is located south of the river. The neighborhood-level locations of the melioidosis cases from 2008–2013 were retrieved from the melioidosis databases at the Centers for Disease Control (CDC) in Taiwan. This database only showed weekly incidences after 2011. The Central Weather Bureau (Taiwan) collected the rainfall (mm), wind speed (m/sec) and wind direction (60° interval) per hour data at a recording station in the endemic area. According to the monthly data, which indicated that rainfall (>200 mm) first appeared in the month of the last typhoon alarm, the rainy season was defined as July to September of 2012 and July to October of 2013 (S1 Fig).

### Soil sampling

Random sampling or fixed-interval soil sampling strategies were used. Random sampling was performed every month from January 2011 to December 2013. According to geographic information system (GIS) analysis, the entire area under study (72 km^2^) was divided into 36 grids (*ca*. 2 km^2^ per grid). Soil was sampled from a depth of 30–60 cm from the land surface in cropped fields, wetted but non-cropped fields, urban grounds (empty land in urban) or urban green areas, with 2 or 3 holes per grid (Fig 1). The soil was sampled from the same sample hole location from each grid whenever possible. Of the sampling sites, one with a high *B*. *pseudomallei* isolation rate was chosen to perform soil sampling at semimonthly intervals from May 2012 to April 2014 using a fixed-interval method. The area (25 m^2^) in this site was evenly divided into 25 grids (1 m^2^ per grid) to sample holes that were evenly distributed at a distance of 1 m from one another. Soil was sampled from the superficial (<10 cm), shallow (30 cm) and deep (60 cm) layers of each hole. The digger was disinfected with 70% alcohol between soil collections. Approximately 50–100 g of soil was collected and immediately sent to the laboratory for analysis. The moisture content (water content) of the soil was measured by subtracting the weight of the completely dry soil from the weight of the hydrated soil.

### Air sampling

Fig 1 shows the locations of air sampling sites. From May 2012 to April 2014, aerosols at each air-sampling site were filtered on a sterile Teflon membrane by pumping (20 L/min) for 8 hours twice per month. With the exception of this semimonthly collection, the air samples were collected from Site G and Site I every day from June to November, and every 2 or 3 days in December and from January to May.

### *B*. *pseudomallei* isolation and identification from air and soil

To cultivate *B*. *pseudomallei* from the air, 30 mL of sterile modified Ashdown’s liquid media (4% glycerol, 5 mg/L crystal violet, 50 mg/L neutral red and 4 mg/L of gentamicin in 5 g/L trypticase soy broth) was inoculated with air (25 mL/min) in an impinging bottle (ACE Glass Inc., Vineland, NJ, USA) for 8 hours over 2-day intervals from July 2013 to October 2013. To avoid the loss of liquid media by evaporation in the bottle, the liquid media was re-supplemented with additional media to a total volume of 30 mL every hour. After an 8-hour collection period, the 30 mL of liquid media was transferred into 50 mL of selective Ashdown’s broth (same formula as a modified Ashdown’s media, except with 10 g/L of trypticase soy broth) in a 250-mL flask for incubation at 42°C. On days 0, 1, 3, and 7, the cultures were streaked on Ashdown’s media.

To isolate *B*. *pseudomallei* from the soil, approximately 5 g of soil was placed into 50 mL of Ashdown’s broth, and the soil particles were dispersed by sonication. The cultures were incubated at 37°C and streaked on Ashdown’s media on days 0, 1, 3, and 7. After incubation at 37°C, the typical wrinkled colonies were selected and identified using biochemical test profiles (API system; bioMérieux), and the presence of the specific amplicons of the 16S RNA gene (243 and 405 bp) and flagellar gene (267 bp) was verified [14]. All primers used in this study to amplify specific targets by PCR are listed in S1 Table.

### Detection of *B*. *pseudomallei*-specific DNA in aerosols

After harvesting aerosols, the Teflon membrane was washed with phosphate-buffered saline (PBS) using sonication, and the particles were precipitated by centrifugation (4500x g, 10 min). Total DNA was extracted using a QIAamp DNA Mini kit (QIAGEN, GmbH, Hilden, Germany). *B*. *pseudomallei*-specific DNA was targeted with a 115-bp stretch in ORF2 of the type III secretion system gene using a specific primer set for amplification (S1 Table) [24]. ORF2-positive samples were analyzed to quantify the concentration of the specific gene using a 7900 HT Fast Real-Time qPCR system (Applied Biosystems Inc., Foster City, CA, USA). In each qPCR reaction, a solution containing 100 CFU/mL of *B*. *pseudomallei* was used as a positive control, and a negative control (sterile water) was processed in parallel. The calibration curve was generated with standard target DNA (purchased from Mission Biotech Inc., Taipei, Taiwan) using ten-fold serial dilutions. Under our study conditions, the calibration curve ranged from -3.6 to -3.1 (the PCR efficiency was 90–110%), and the linear regression coefficient (*r^2^*) was 0.998. The lower detection limit was 6.94 copies/m^3^, and approximately 1 copy/μl in the real-time qPCR assay was equal to 5.5 CFU/μl [24].

### Molecular typing

Pulsed-field gel electrophoresis (PFGE) analysis and multilocus sequence typing (MLST) were performed according to previously published protocols [25]. Briefly, PFGE was performed using a CHEF Mapper System (Bio-Rad, Hercules, CA, USA) after SpeI-digestion. A field angle of 120° and a voltage gradient of 6 V/cm were utilized for field electrophoresis. DNA from the *Salmonella enterica* serotype Braenderup H9812 (ATCC BAA-664; provided by the CDC, Atlanta, GA, USA) was used as a molecular size marker. The PFGE patterns were imaged with BioNumerics software (Applied Maths, Sint-Martens-Latem, Belgium, China). For MLST, 7 of the housekeeping genes (*ace*, *gltB*, *lepA*, *lipA*, *nark*, *ndh* and *gmhD*) were amplified and sequenced (S1 Table). The alleles at each locus were assigned by comparing them with the sequences on the website (http://bpseudomallei.mlst.net/). The ST types were determined using the allelic profile database. The novel ST1354 type in this study was registered in the *B*. *pseudomallei* database because new alleles in the *gmhD* and *lepA* gene sequences were identified.

### Statistical analysis

Statistical analysis was performed using SPSS (version 17.0). Ranges, means and standard errors were used to describe specific DNA concentrations, isolation rates and PCR positivity. The *B*. *pseudomallei*-specific DNA concentrations in different air-sampling sites were compared using Student’s t-test. Spearman’s correlation or linear regression analysis was used to compare the monthly isolation rate and the average soil moisture content per month as well as the monthly isolation rate and the average PCR positivity per month. To test the relationship between disease incidence and the appearance of the specific DNA concentration at different sites, we added an auto-regression model to extract the effect of autocorrelation, which was controlled from lag 1 to lag 6 [26], as follows: incidence = DNA concentration in Site G (X_1_) + DNA concentration in Site I (X_2_) + interaction (X_3_, DNA concentration in Site G *vs* DNA concentration in Site I) + auto-regression of incidence from lag 1 (X_4_) to lag 6 (X_9_) (extract the effect of autocorrelation) per week. In the pre-test, no correlation was found if the time lag was set as per week. Additionally, none of the variables such as rainfall, wind direction and wind speed correlated with the DNA concentration if added into this equation. The daily data was thus used to assess the correlation between the *B*. *pseudomallei*-specific DNA concentration in aerosols and climatic factors such as rainfall, wind speed and wind direction using multivariable regression analysis. Because the aerosols were collected every 2 or 3 days in the dry season (January to May and December), the average data from the former and latter days were employed as the empty data. However, the specific DNA concentration was recognized as zero in most cases during the dry season because no specific PCR product was detected. Multivariable regression analysis was based on the following equation: DNA concentration = rainfall (X_1_, mm) + wind direction (X_2_, times) + wind speed (X_3_, m/sec) + interaction (X_4_ rainfall *vs* wind speed; X_5_, rainfall *vs* wind direction; X_6_, wind direction *vs* wind speed) + auto-regression of DNA concentration from Lag 1 (X_7_) to Lag 6 (X_13_). The time lag was set from 0 to 5 day. The level of significance was set at 0.05.

### Accession numbers

The nucleotide sequences obtained in this study are available at the National Center for Biotechnology Information (NCBI) under the following accession numbers: *fliC* (U82287.1), 16S RNA (AY305818) and ORF2 of the type III secretion system (AF074878). Nucleotide sequences of the 7 housekeeping genes (*ace*, *gltB*, *lepA*, *lipA*, *nark*, *ndh* and *gmhD*) are included in the *B*. *pseudomallei* database (http://bpseudomallei.mlst.net/).

## Results

### Geographical distribution of *B*. *pseudomallei* across the endemic area

**Fig 2:**
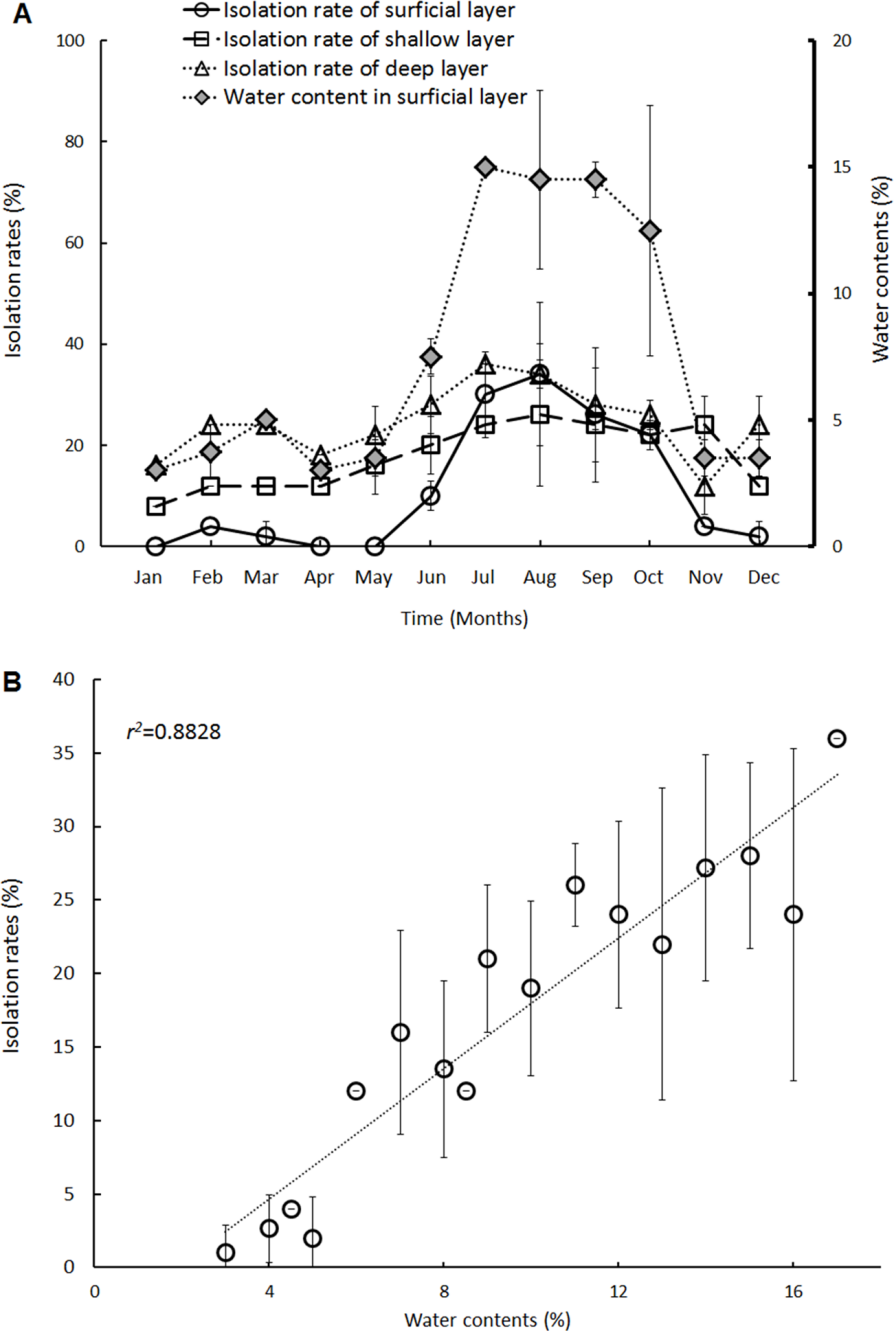
The isolation rate distribution and correlations between the isolation rate and moisture content. The soil (<10 cm, superficial layers, circle; 30 cm, shallow layer, square; 60 cm, deep layer, hollow triangle) was sampled in a vertical distribution. The average monthly isolation rates of *B*. *pseudomallei* from each layer are shown. The moisture (water) contents in the superficial layer are labeled with grey diamonds (A), and the isolation rates are shown for the indicated moisture contents (B).

From 2008 to 2013, 75 melioidosis cases were clustered in the Zoynan district, which was defined as an endemic area for melioidosis in Taiwan [21]. In this endemic area, approximately 90% (n = 68) of the melioidosis cases were distributed in the south, which is referred to as the core region (Fig 1). We prospectively investigated the geographical distribution of *B*. *pseudomallei* in soil in the core region and its surroundings (total area *ca*. 72 km^2^) over 3 years (2011–2013). During the investigation, *B*. *pseudomallei* was not found in urban grounds or urban green areas. Additionally, *B*. *pseudomallei* was not widely distributed in rural areas; rather, it was isolated from cropped fields (n = 4) or wetted but non-cropped fields (n = 2) (S2 Table). All positive isolation sites were located in the northern or northwestern outskirts (*ca*. 300°-360°) of the core region. A northwestern site that appeared to have a high isolation rate (20.8±7.22% per year) was selected to survey the vertical distribution of *B*. *pseudomallei* from the land surface to deep soils. In the superficial layers (<10 cm), the monthly isolation rates of *B*. *pseudomallei* were 22±2.8%-34±2.8% from July to October and 0±0%-10±2.8% during the other months. In the shallow or deep layers, the isolation rates fluctuated, ranging from 8±0% to 26±14% in shallow soil and from 12±2.8% to 36±8.5% in deep soil (Fig 2A). Note that *B*. *pseudomallei* thrives in moist soil [27–28]. In this study, the isolation rates in soils correlated with the soil moisture content (Fig 2B) (*r^2^* = 0.8828). In Taiwan, typhoons that carry heavy rainfalls and result in floods usually occur from July to October (typhoon season). However, in 2012, the last typhoon alarm was in September (refer to S1 Fig). On average, 1.33 invasive typhoons occurred per month during the typhoon seasons from 2012 to 2013. The average moisture content in the superficial layers was relatively high from July to October, which corresponded to the typhoon season (Fig 2A).

### Geographical distribution of aerosols contaminated with *B*. *pseudomallei* in endemic areas

**Fig 3:**
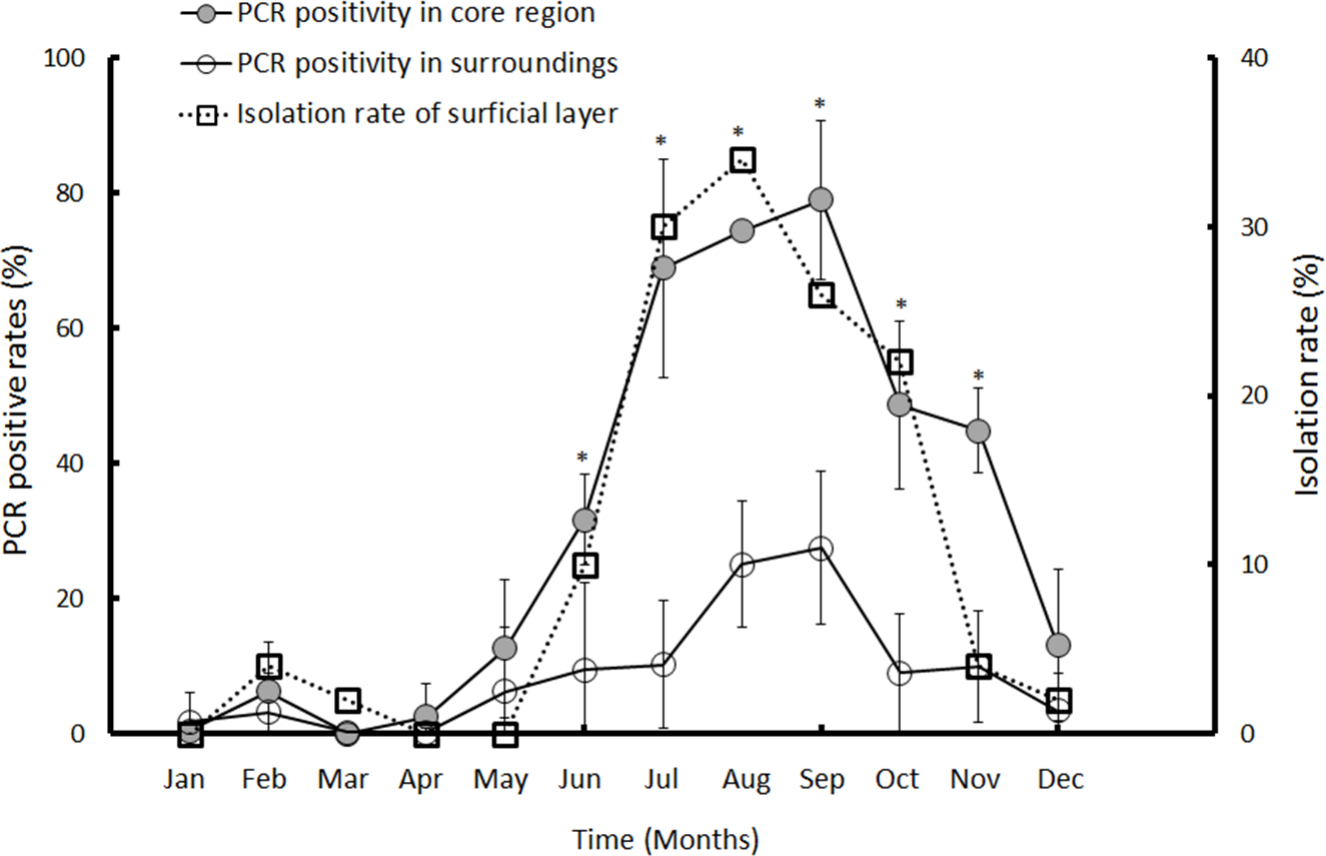
PCR positivity rates and the correlation between PCR positivity and the *B*. *pseudomallei* isolation rate. Air particles were collected from different sites (See Fig 1). The average PCR positivity rates per month at Sites D-G (core region, black circle) and Sites A-C and H-L (surroundings, hollow circle) are shown. The significant difference (*, p<0.05) of monthly PCR positivity between the core region and surroundings is shown. The isolation rates of *B*. *pseudomallei* (hollow square) in the superficial layer of a northwestern cropped field are shown.

We examined the prevalence of aerosols positive for *B*. *pseudomallei*-specific DNA in the core region. In a 2-year prospective investigation (May 2012 to April 2014), 29.2±0%-40.7±16.6% of aerosols in the core region were positive for *B*. *pseudomallei*-specific DNA per year (Sites D-G), but the rate ranged from 7.3±1.4%-11.4±1.5% per year in the surrounding areas (Sites A-C and Sites H-L) (S3 Table). As shown in Fig 3, the monthly PCR-positive rates for aerosols collected from the core region significantly increased from June to November compared with the rates estimated from the surrounding areas (t-test, p<0.05). In particular, the increase in PCR-positive rates for aerosols from the core region correlated with the isolation rates from the superficial layers of the northwestern cropped field (Spearman’s correlation test, α = 0.859, p<0.001).

**Fig 4:**
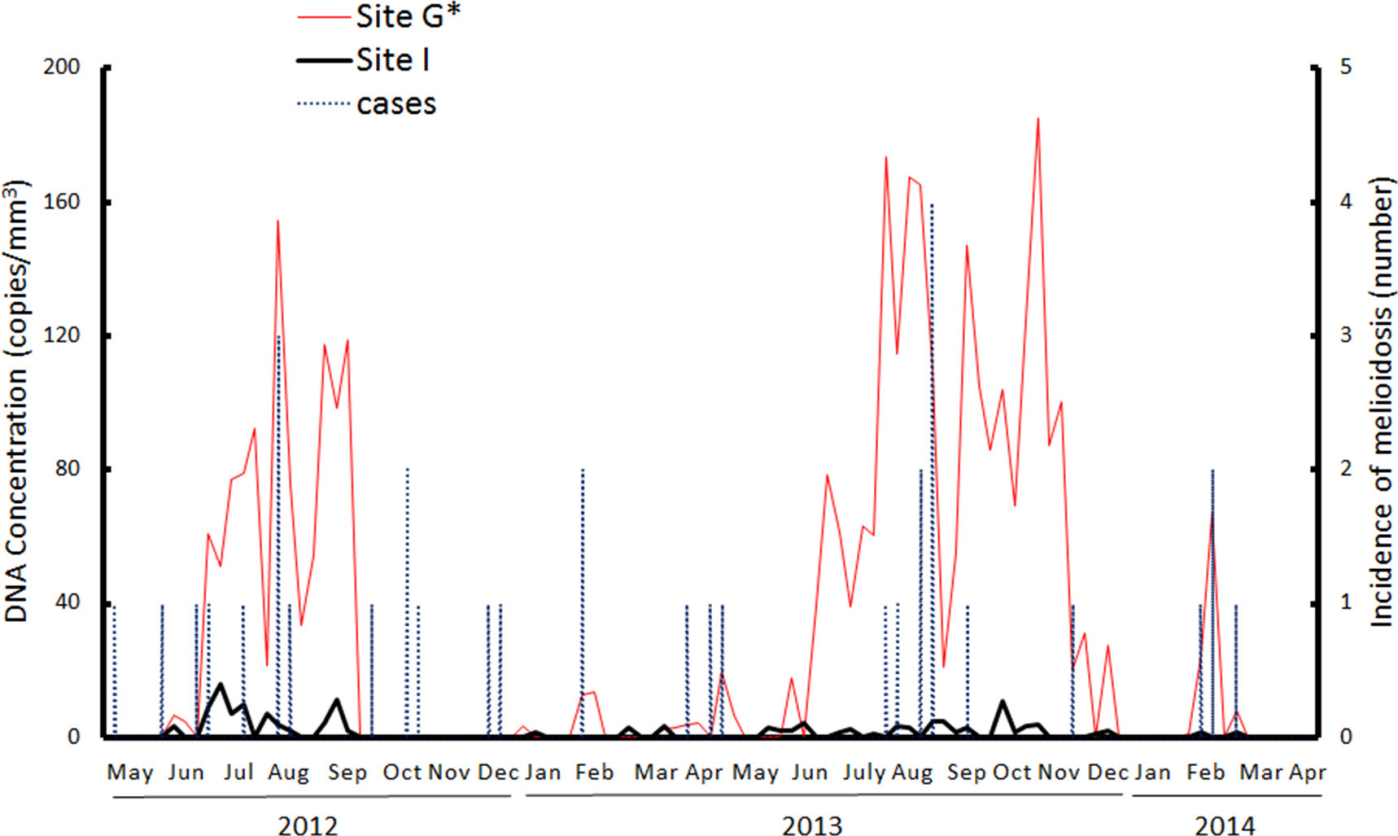
*B. pseudomallei*-specific DNA concentration and melioidosis cases. From May 2012 to April 2014, the *B*. *pseudomallei*-specific DNA concentration was measured from the total air particles collected from Sites G (red line) and I (black line) (location see Fig 1). The melioidosis cases (blue dotted line) are shown. The symbol (*) indicates the correlation between contaminated aerosols and the melioidosis incidence.

Sites G and I were best suited for comparisons to determine an association between northwesterly winds and the appearance of contaminated aerosols because hills, which obstruct wind, likely interfered with aerosol transmission from the north to the south (Fig 1). In a parallel study conducted from May 2012 to April 2014, the concentration of *B*. *pseudomallei*-specific DNA at Site G was significantly higher than that at Site I (t-test, p<0.05). During this period, changes in the *B*. *pseudomallei*-specific DNA concentration in Site G were related to the melioidosis incidence (lag 0 week, p<0.05; lag 1 week, p = 0.067). The low concentration of *B*. *pseudomallei*-specific DNAs at Site I was responsible for the lack of correlation between the DNA concentration and the disease incidence (Fig 4). However, these aerosols contaminated with *B*. *pseudomallei*-specific DNA were potentially dangerous because the pathogen was isolated from approximately 6.7% (4/60) of aerosols at Site G. *B*. *pseudomallei* was not isolated from the aerosols at Site I, which contained low levels of pathogen-specific DNA (Table 1).

**Table 1:** Proportion of positive cultures from aerosols in different sites.

Location	Period of air collection	Sample sizes	*B*. *pseudomallei* growth (%)	Method
Site G	Jul-Oct, 2013	60	4 (6.7%)	Impinging
Site I	Jul-Oct, 2013	60	0 (0%)	Impinging

### Relationship between contaminated aerosols and wind direction

To assess whether the *B*. *pseudomallei*-contaminated aerosols were carried by northwesterly winds from cropped fields, we defined *B*. *pseudomallei*-specific DNA concentration in aerosols as an independent variable and wind direction, wind speed and rainfall as dependent variables. According to a multilevel analysis with a time series and auto-regression, the *B*. *pseudomallei*-specific DNA concentration in aerosols was positively correlated with the wind direction (300°-360°, northwesterly to northern wind) (p<0.05) and wind speed of the northwesterly wind (p<0.05) in site G (Table 2). Other wind angles were not correlated with the pathogen-specific DNA. The contaminated aerosols likely were not correlated with the northwesterly wind or the wind speed as the time lag occurred because the aerosols were scattered by winds from other directions on the next day. At site I, hills interfere with the path of northwesterly wind. Therefore, the *B*. *pseudomallei*-specific DNA concentration was not correlated to the wind direction or speed if the contaminated sources were located in a northwestern cropped field (Table 2).

**Table 2:** Association between the *B. pseudomallei*-specific DNA concentration and multiple variables (rainfall, wind direction [WD] and wind speed [WS]) with time lag.

Category		Variables in^#^					
		Site G			Site I		
(angles)	Lag (d)	Rainfall	WD	WS	Rainfall	WD	WS
301°	0		p<0.05	p<0.05			
/	1				p<0.05		
360°	2				p<0.05		
	3				p<0.05		
	4						
	5						
241°	0			p<0.05			
/	1				p<0.05		
300°	2		p<0.05		p<0.05		
	3						
	4	p<0.05					
	5						
181°	0						
/	1				p<0.05		
240°	2				p<0.05		
	3			p<0.05			
	4						p<0.05
	5						
121°	0						
/	1						
180°	2		p<0.05				
	3				p<0.05		
	4						
	5			p<0.05			
61°	0						
/	1					p<0.05	
120°	2						
	3						
	4						
	5						
1°	0						
/	1				p<0.05		
60°	2				p<0.05		
	3				p<0.05		
	4	p<0.05					
	5						

^#^ significance to positive correlation

### Molecular association among soil, aerosol and human isolates

To determine whether the pathogen was transmitted from contaminated soil to humans as an aerosol, 47 isolates collected from patients (n = 21) who dwelled in core regions, from aerosols (n = 4) collected from the core region, and from soils (deep layer, n = 7; shallow layer, n = 7; superficial layer, n = 8) collected from northwestern cropped fields were genetically typed using MLST and PFGE. According to the MLST analysis, 6 ST types (ST58, ST704, ST834, ST1001, ST1115 and ST1354) appeared among the strains (S4 Table). Both ST58 and ST1001 were commonly identified in environmental isolates, whereas only ST58 was identified in human isolates. According to PFGE analysis, the soil isolates were mainly categorized into Groups Ia, Ib, Ic and II, and the human isolates were only grouped into Ia (S2 Fig). All analyzed human or aerosol isolates were categorized as PFGE Type Ia (or ST58 by MLST) (Table 3). The molecular type PFGE Ia (ST58) linked the soil isolates in the superficial layer to aerosols and human samples.

**Table 3:** Summary of ST types and PFGE patterns of *B. pseudomallei*.

Sources	Year	Number	Proportions (%) of ST types (or PFGE patterns)					
		n	58 (Ia)	834 (Ia)	1354 (Ib)	704 (Ic)	1001 (II)	1115 (II)
Human	2010	8	100					
	2011	5	100					
	2012	4	100					
	2013	2	100					
Aerosol	2013	4	100					
Soil								
Superficial	2012	4	50	25			25	
layer	2013	4	75				25	
Shallow	2012	4	25				50	25
layer	2013	3	33				67	
Deep	2012	3	33	33			33	
layer	2013	4	50		25	25		

**Fig 5:**
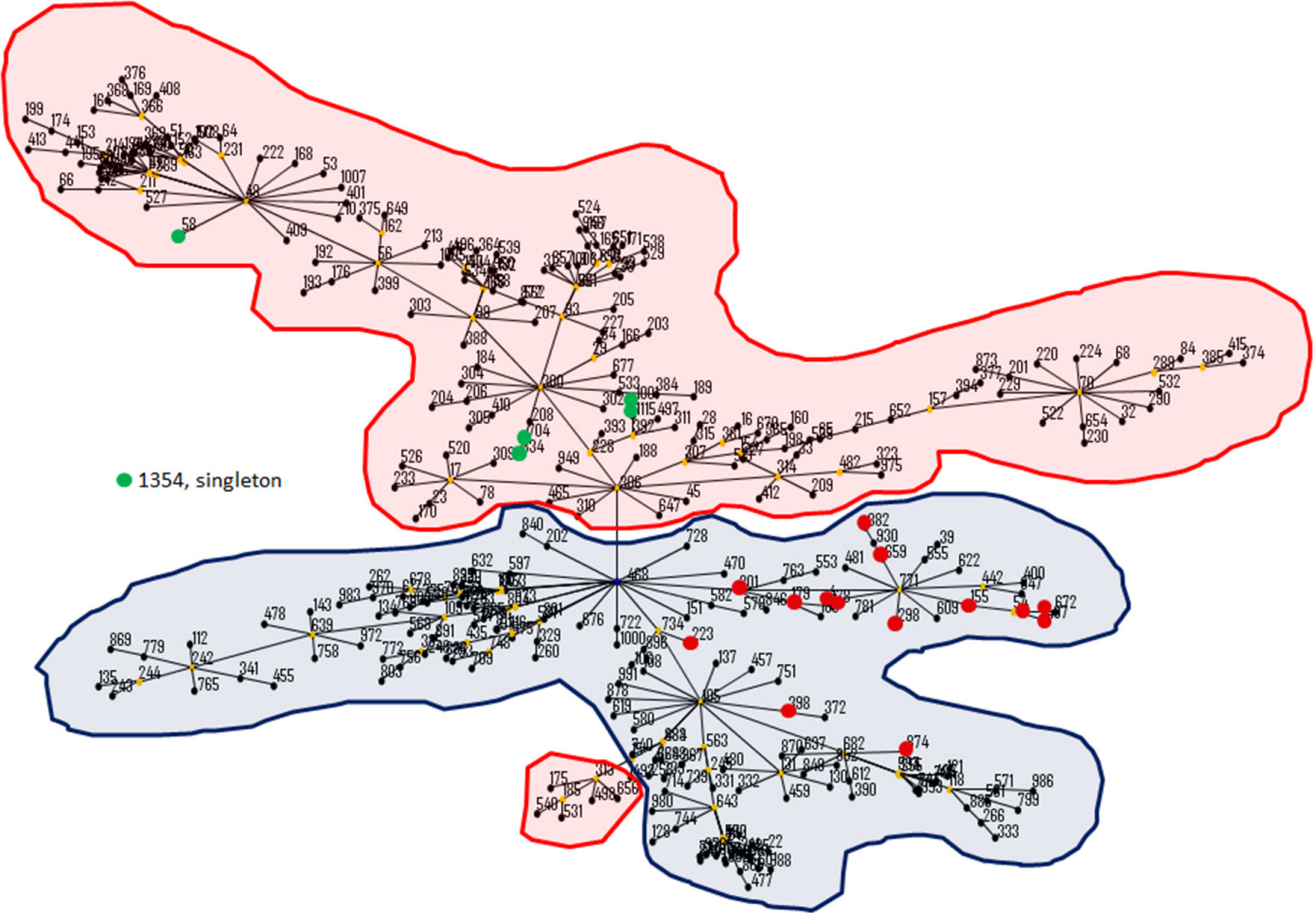
Phylogenetic relatedness of *B. pseudomallei* strains. The distinct ST types (Taiwanese ST types, shown as green circles; Thai ST types, shown as red circles or localized at the region in pink; Australian ST types, localized at the region in blue) that were identified using eBurst (v3) analysis are shown. ST1354 (singleton) is a novel ST type, shown on the left.

*B*. *pseudomallei* isolates worldwide are reportedly categorized into Thai and Australian clones [29]. According to the phylogenetic analysis, except for a single ST1354 isolate, all Taiwanese isolates in this study were evolutionally similar to Thai clones (Fig 5).

## Discussion

Climatic factors such as rainfalls, typhoons or cyclones are trigger factors that contribute to the melioidosis incidence in endemic areas such as Thailand, Australia, Singapore and Taiwan [8–9,11,16]. Of these factors, heavy rainfall, mainly due to the typhoon season, was the predominant factor in Taiwan [11]. Heavy rainfalls likely resulted in flooding that allowed the soil to be saturated by water. In this study, the *B*. *pseudomallei* isolation rate was correlated with increased moisture content in the superficial layers of a cropped field. Water served as a vehicle to spread *B*. *pseudomallei* contamination. [7,21]. Drinking water, soil and dust have been identified as potential sources of dissemination [21,30–31]. However, assessing whether humans are exposed to these contaminants *via* direct contact, ingestion or inhalation is difficult. In a previous study, we developed a qPCR method and used it to determine that the *B*. *pseudomallei*-specific DNA level was increased during the typhoon (rainy) season [24]. In this study, we integrated data for the geographical distribution of *B*. *pseudomallei* in soil, the incidence of melioidosis in this endemic area, the appearance of *B*. *pseudomallei*-specific DNA in the core region, the role of climatic factors and the molecular associations among soil, aerosol and human isolates. Our results indicate that the concentration of *B*. *pseudomallei*-specific DNA in aerosols was associated with the incidence of melioidosis and the appearance of a northwesterly wind during the typhoon season. By tracing this wind direction, *B*. *pseudomallei* was steadily isolated from the superficial layers of a cropped field during the typhoon season. PFGE Type 1a *B*. *pseudomallei* isolates (ST58, by MLST), the prevailing type of clinical melioidosis isolate in Taiwan [25], were isolated from aerosols. Thus, the melioidosis transmission mode in this endemic area includes environmental aerosol inhalation. This evidence is the first to identify the transmission mode from contaminated soils to aerosols and/or to patients. Approximately 70% of melioidosis patients presented with pulmonary inflammation in an outbreak in Taiwan [32].

The natural habitats of *B*. *pseudomallei* include moist, slightly acidic and nutrient-rich soil [27,33], the rhizosphere, roots and aerial parts of grasses [34], and water [13,35]. These bacterial niches are usually located in cropped fields or non-disturbed lands in rural areas, although several physical, chemical or biological antagonists affect the geographical distribution of *B*. *pseudomallei* in soil [27,33,36]. Thus, *B*. *pseudomallei* is usually isolated from rural areas [9,21,37–38] and not from urban ground or urban green areas (in this study). However, melioidosis cases are often clustered in the city and not in rural villages. The evidence of *B*. *pseudomallei*-contaminated aerosols indicates that patients in the city may have inhaled environmental aerosols generated from rural areas; however, the possibility that patients acquired melioidosis from a subcutaneous inoculation of contaminants during travel cannot be excluded. Air dispersion and climate conditions such as atmospheric stability (sunshine or rainfall), wind speed and wind direction predominantly contribute to the distance that contaminants are carried downwind (please refer to the Gaussian equation for the air dispersion model: http://www.csun.edu/~vchsc006/469/gauss.htm). The pathogen of Legionnaire’s disease can travel up to 6 km by air dispersion [39]. In this study, a cropped field harboring a high *B*. *pseudomallei* isolation rate was located 5 km away from the core region, with the clusters of melioidosis cases in the northwest direction. Porous wetted surfaces, which likely comprised different forms of soils such as clay and sand, could generate aerosols in response to impact from equivalently sized rain droplets [40]. Winds that blow across the surface of porous media can carry tiny droplets or solid particles (<50 mm) for several thousands of kilometers [40]. Thus, in addition to agricultural activity, extreme climate conditions such as typhoons could generate numerous outdoor aerosols [39,41–42]. The correlation between the appearance of *B*. *pseudomallei*-specific DNA in aerosols and strong wind speeds with wind angles ranging from 300°-360° (northwesterly wind) indicated that cropped fields represent an important source of contaminants.

Rainfall in Taiwan mainly comprises plum rain (April to mid-June), which is similar to April showers, and typhoons (July to October), which usually involve heavy rains. Our data indicate that the moisture content in the soil surface was approximately 3–7.5% from May to June but 13–15% from July to October. Plum rain usually drizzles, although it can persist for two weeks; thus, the water that accumulates on the soil surface is not sufficient to support the growth or dissemination of *B*. *pseudomallei*. However, rainfall intensifies during the typhoon season, and sudden rains can result in flooding. Thus, strong winds generated the *B*. *pseudomallei*-contaminated aerosols. Our regression model indicates that both wind direction and wind speed contributed to the presence of specific *B*. *pseudomallei*-ORF2 DNA at the downwind site (Site G) if lag day 0 was defined as the start of the high northwesterly wind speed. However, rainfall was correlated with *B*. *pseudomallei*-specific DNA levels if the lag day was set to 1–3 days at Site I. This difference likely arose because hills obstructed the wind at Site I. Additionally, intense rainfall also eliminated the aerosols from the air, although raindrops that hit the ground could also generate aerosols [40]. We and other groups have indicated that the melioidosis incidence was correlated with increased rainfall when the different time lag was defined [6,11]. At Site I, as wind direction and wind speed did not contribute to presence of *B*. *pseudomallei*-specific DNA, contaminated aerosols were correlated with rainfall at a time lag of 1–3 days.

From 2006–2011, 5 melioidosis outbreaks occurred in Taiwan [25]. Although melioidosis cases were clustered in the core region due to a high concentration of *B*. *pseudomallei*-specific DNA during the typhoon season, the areas surrounding the core region remained at risk because the concentration of *B*. *pseudomallei*-specific DNA was not directly correlated with the number of viable *B*. *pseudomallei*. Specifically, contaminated aerosols could be transmitted to areas thousands of kilometers away [40]. However, Site G in the core region was suitable to monitor the appearance of contaminated aerosols and the incidence of melioidosis. We previously reported that *B*. *pseudomallei* ST58 was an epidemic strain among the clinical isolates collected from 2006 to 2011 [25]. In this study, *B*. *pseudomallei* ST58 remained the predominant strain among melioidosis patients residing in the endemic area from 2012 to 2014. In particular, *B*. *pseudomallei* ST58 was also prevalent in environmental strains isolated from cropped or wetted but non-cropped fields and from aerosols. Physicians should monitor the pattern of epidemic strains if the molecular types of *B*. *pseudomallei* change and should be concerned with the airborne transmission of melioidosis in Taiwan.

## Supporting Information

S1 Fig Rainfall and typhoon alarms. The monthly rainfall (mm, solid line) from January 2012 to March 2014 and the average 9-year monthly rainfall data (dotted line) from 2006 to 2014 are shown. Two peaks of rainfall per year were due to plum rain (April showers) and typhoons (heavy rains). After the typhoons, the appearance of stagnant pools of water on farmland was recorded (grey line). Plum rains did not result in stagnant pools of water. The black point indicates a typhoon alarm from 2012 to 2013. In both years, typhoons causing heavy rainfalls appeared in July. The last typhoon alarms occurred in September 2012 and October 2013. In this study, the rainy (typhoon) seasons (black line) were defined from July to September in 2012 and from July to October in 2013.
(TIF)

S2 Fig Phylogenetic analysis of PFGE patterns. With 90% similarity, the *B*. *pseudomallei* isolates (S, soil isolates; H, human isolates; A, aerosol isolates) in this study were organized into Groups I and II. Group I was subdivided into Groups Ia, Ib and Ic. The ST types of each isolate are shown at the bottom of this figure.
(TIF)

S1 Table Targets, primer sequences and PCR conditions. (DOCX)

S2 Table Location and isolation rate of sample sites. (DOCX)

S3 Table The location and PCR positive rates of aerosols at air sampling sites. (DOCX)

S4 Table Strain information, ST types and alleles of *B. pseudomallei* isolates. (DOCX)
