# Supplementary figures and images for: Airborne Transmission of Melioidosis to Humans from Environmental Aerosols Contaminated with B. pseudomallei
Source: PLoS Negl Trop Dis. 2015 Jun 10;9(6):e0003834. doi: 10.1371/journal.pntd.0003834 (PMC4462588; doi:10.1371/journal.pntd.0003834)

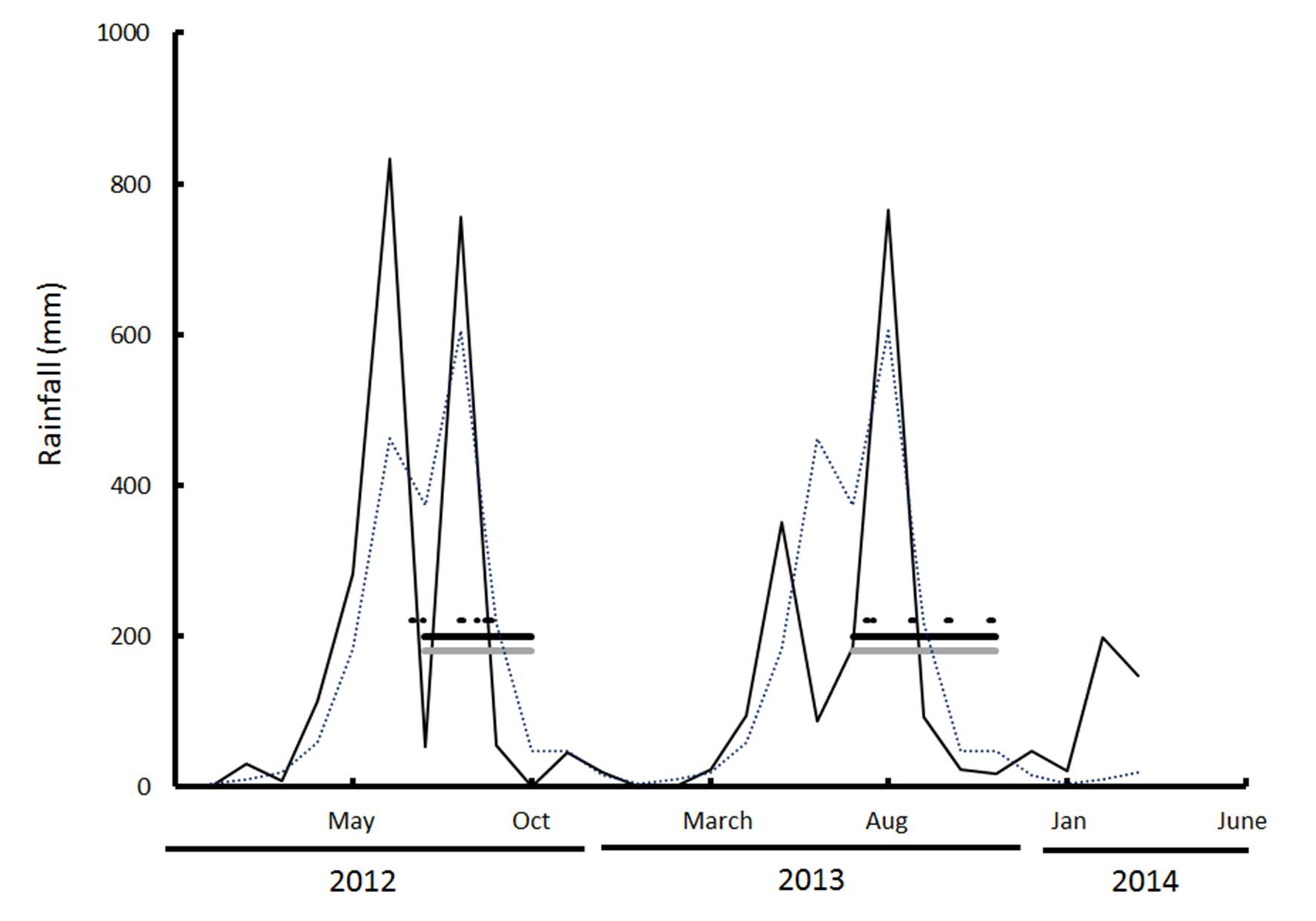

Supplement: S1 Fig — The monthly rainfall (mm, solid line) from January 2012 to March 2014 and the average 9-year monthly rainfall data (dotted line) from 2006 to 2014 are shown. Two peaks of rainfall per year were due to plum rain (April showers) and typhoons (heavy rains). After the typhoons, the appearance of stagnant pools of water on farmland was recorded (grey line). Plum rains did not result in stagnant pools of water. The black point indicates a typhoon alarm from 2012 to 2013. In both years, typhoons causing heavy rainfalls appeared in July. The last typhoon alarms occurred in September 2012 and October 2013. In this study, the rainy (typhoon) seasons (black line) were defined from July to September in 2012 and from July to October in 2013. (TIF) [file pntd.0003834.s001.tif]

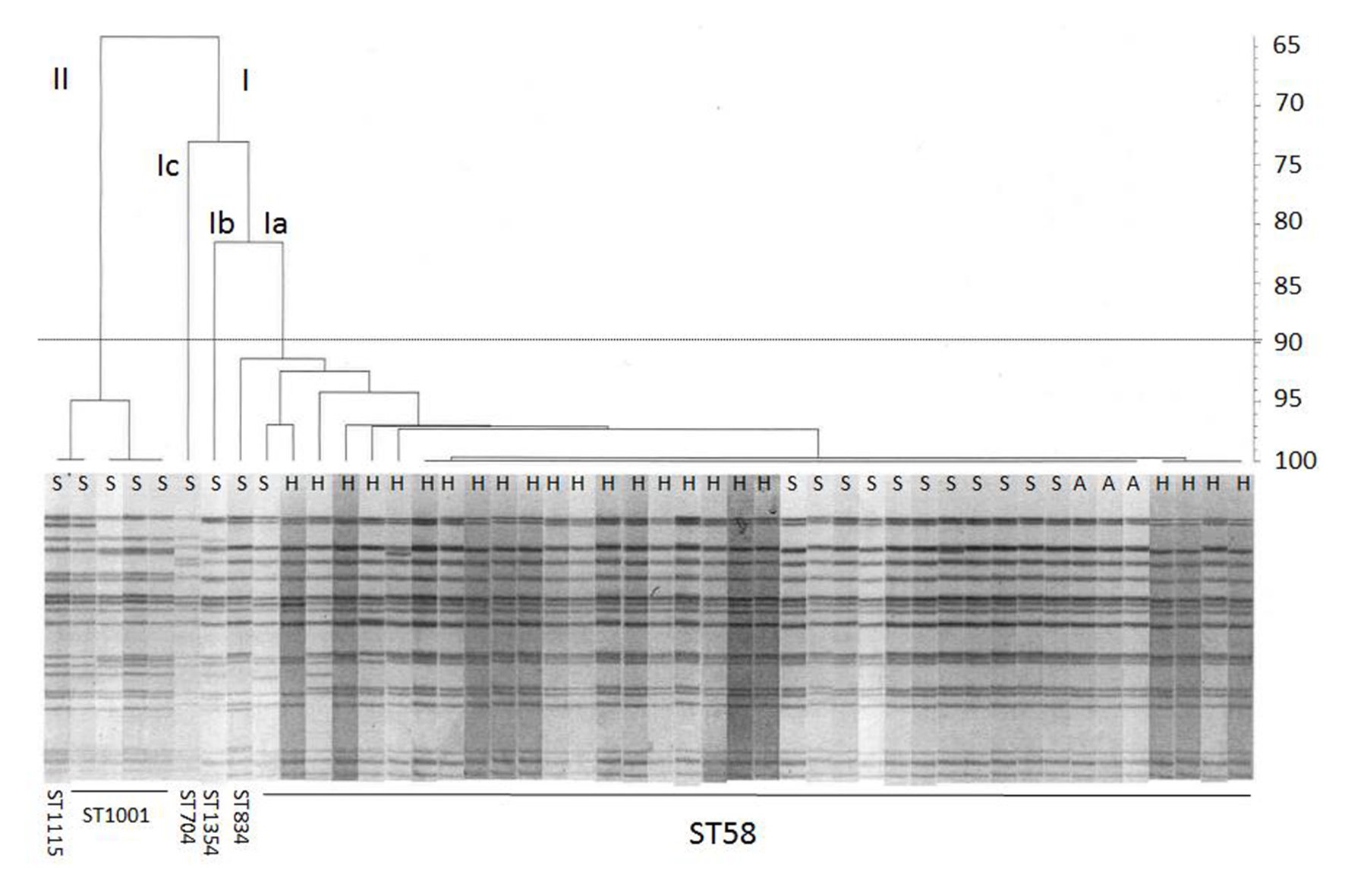

Supplement: S2 Fig — With 90% similarity, the B. pseudomallei isolates (S, soil isolates; H, human isolates; A, aerosol isolates) in this study were organized into Groups I and II. Group I was subdivided into Groups Ia, Ib and Ic. The ST types of each isolate are shown at the bottom of this figure. (TIF) [file pntd.0003834.s002.tif]
