# Supplementary material for: Airborne Transmission of Melioidosis to Humans from Environmental Aerosols Contaminated with B. pseudomallei
Source: PLoS Negl Trop Dis. 2015 Jun 10;9(6):e0003834. doi: 10.1371/journal.pntd.0003834 (PMC4462588; doi:10.1371/journal.pntd.0003834)
Supplement: S1 Table — (DOCX) [file pntd.0003834.s003.docx]

**S1 Table, Targets, primer sequences and PCR conditions**

| Target | Primer sequence (5' -> 3') | Size  (bp) | PCR condition |
| --- | --- | --- | --- |
| *fliC* | F: CTGTCGTCGACGGCCGTG | 267 | Denature, 94°C, 1 min; Anneal, |
|  | R: ATTGTTGACGTCGCGAG |  | 60°C, 30 sec; Extend, 72°C, 1 min |
| 16S | F: CGGCAGCGCGGGCTTCGG | 243 | Denature, 94°C, 1 min; Anneal, |
| RNA | R1: TGTGGCTGGTCGTCCTCTC | 405 | 60°C, 30 sec; Extend, 72°C, 1 min |
|  | R2: CACTCCGGGTATTAGCCAGA |  |  |
| *orf2* | F: CGTCTCTATACTGTCGAGCAATCG | 115 | Denature, 95°C, 15 sec; Anneal, |
|  | R: CGTGCACACCGGTCAGTATC |  | 59°C, 15 sec; Extend, 72°C, 30 sec |
| *ace* | F: GAATCGCCTTCACCATGTC | 617 | Denature, 94°C, 30 sec; Anneal, |
|  | R: CGGCGCTTCTCAAAACGATA |  | 62°C, 30 sec; Extend, 72°C, 1 min |
| *gmhD* | F: GCAGTTCCTGTATGCGTC | 559 | Denature, 94°C, 30 sec; Anneal, |
|  | R: GAAGCACTGGTACTTGCC |  | 63°C, 30 sec; Extend, 72°C, 1 min |
| *ndh* | F: AGTCGCGACGTTCTACAC | 567 | Denature, 94°C, 30 sec; Anneal, |
|  | R: CGAGTTGCAGACGAGATA |  | 62°C, 30 sec; Extend, 72°C, 1 min |
| *gltB* | F: ACGCTCGCGATCGCGATGAA | 647 | Denature, 94°C, 15 sec; Anneal, |
|  | R: TTCAGCACGAGCGTCTGCTG |  | 58°C, 30 sec; Extend, 72°C, 1 min |
| *nark* | F: CTACTCGTGCGCTGGGAT | 642 | Denature, 94°C, 30 sec; Anneal, |
|  | R: GACGATGAACGGCACCCA |  | 62°C, 30 sec; Extend, 72°C, 1 min |
| *lepA* | F: CACATCGACCACGGC | 624 | Denature, 94°C, 30 sec; Anneal, |
|  | R: GAGCACGTCCTCGAC |  | 61.5°C, 30 sec; Extend, 72°C, 1 min |
| *lipA* | F: GGCACCGCGACGTTCATG | 462 | Denature, 94°C, 30 sec; Anneal, |
|  | R: GACCATCAGGCCCGATTTCG |  | 68°C, 30 sec; Extend, 72°C, 1 min |
