# Supplementary material for: Airborne Transmission of Melioidosis to Humans from Environmental Aerosols Contaminated with B. pseudomallei
Source: PLoS Negl Trop Dis. 2015 Jun 10;9(6):e0003834. doi: 10.1371/journal.pntd.0003834 (PMC4462588; doi:10.1371/journal.pntd.0003834)
Supplement: S2 Table — (DOCX) [file pntd.0003834.s004.docx]

**S2 Table. Location and isolation rate of sample sites**

| Site No. | GPS | Utilization | Isolation rates (%) | | | Average |
| --- | --- | --- | --- | --- | --- | --- |
|  |  |  | 2011 | 2012 | 2013 | Mean ±SD |
|  |  |  | n=24 | n=24 | n=24 |  |
| 1 | 22.7667,120.2387 | Wetted but non-cropped | 0 | 0 | 0 | 0 |
| 2 | 22.7672,120.2598 | Urban ground | 0 | 0 | 0 | 0 |
| 3 | 22.7689,120.2848 | Cropped | 0 | 0 | 0 | 0 |
| 4 | 22.7741,120.2946 | Cropped | 0 | 0 | 0 | 0 |
| 5 | 22.7680,120.3048 | Cropped | 0 | 0 | 0 | 0 |
| 6 | 22.7671,120.3396 | Cropped | 0 | 0 | 0 | 0 |
| 7 | 22.7540,120.2484 | Cropped | 0 | 0 | 0 | 0 |
| 8 | 22.7543,120.2666 | Cropped | 0 | 0 | 0 | 0 |
| 9 | 22.7459,120.3098 | Cropped | 4.2 | 0 | 8.3 | 4.16±4.15 |
| 10 | 22.7587,120.3363 | Cropped | 0 | 0 | 0 | 0 |
| 11 | 22.7390,120.2581 | Cropped | 0 | 0 | 0 | 0 |
| 12 | 22.7406,120.2742 | Cropped | 12.5 | 25 | 25 | 20.8±7.22 |
| 13 | 22.7457,120.2839 | Cropped | 29.1 | 25 | 8.3 | 20.8±11.02 |
| 14 | 22.7435,120.3037 | Cropped | 8.3 | 4.2 | 4.2 | 5.6±2.37 |
| 15 | 22.7433,120.3298 | Cropped | 0 | 0 | 0 | 0 |
| 16 | 22.7416,120.3415 | Cropped | 0 | 0 | 0 | 0 |
| 17 | 22.7255,120.2613 | Wetted but non-cropped | 8.3 | 4.2 | 4.2 | 5.6±2.37 |
| 18 | 22.7272,120.2766 | Urban ground | 0 | 0 | 0 | 0 |
| 19 | 22.7290,120.2950 | Urban ground | 0 | 0 | 0 | 0 |
| 20 | 22.7300,120.3127 | Wetted but non-cropped | 0 | 0 | 0 | 0 |
| 21 | 22.7288,120.3325 | Cropped | 0 | 0 | 0 | 0 |
| 22 | 22.7200,120.2807 | Wetted but non-cropped | 25 | 33.3 | 16.7 | 25±2.8.3 |
| 23 | 22.7145,120.2901 | Urban ground | 0 | 0 | 0 | 0 |
| 24 | 22.7121,120.3243 | Urban ground | 0 | 0 | 0 | 0 |
| 25 | 22.7150,120.3324 | Urban ground | 0 | 0 | 0 | 0 |
| 26 | 22.7096,120.3375 | Urban ground | 0 | 0 | 0 | 0 |
| 27 | 22.6955,120.2860 | Urban ground | 0 | 0 | 0 | 0 |
| 28 | 22.6952,120.2972 | Urban ground | 0 | 0 | 0 | 0 |
| 29 | 22.6979,120.3269 | Urban ground | 0 | 0 | 0 | 0 |
| 30 | 22.6801,120.2826 | Urban ground | 0 | 0 | 0 | 0 |
| 31 | 22.6808,120.3021 | Urban green area | 0 | 0 | 0 | 0 |
| 32 | 22.6830,120.3236 | Urban ground | 0 | 0 | 0 | 0 |
| 33 | 22.6820,120.3407 | Urban ground | 0 | 0 | 0 | 0 |
| 34 | 22.6642,120.2937 | Urban green area | 0 | 0 | 0 | 0 |
| 35 | 22.6637,120.3152 | Urban green area | 0 | 0 | 0 | 0 |
| 36 | 22.6628,120.3411 | Urban green area | 0 | 0 | 0 | 0 |
