# Supplementary material for: Airborne Transmission of Melioidosis to Humans from Environmental Aerosols Contaminated with B. pseudomallei
Source: PLoS Negl Trop Dis. 2015 Jun 10;9(6):e0003834. doi: 10.1371/journal.pntd.0003834 (PMC4462588; doi:10.1371/journal.pntd.0003834)
Supplement: S3 Table — (DOCX) [file pntd.0003834.s005.docx]

**S3 Table. The location and PCR positive rates of aerosols at air sampling sites**

| Site No. | GPS | Positive rates, %  (Total assays, n) | | | Yearly rates, % |
| --- | --- | --- | --- | --- | --- |
|  |  |  | 2012 | 2013 | mean±SD |
| A | 22.7562,120.2462 |  | 10.4 (48) | 8.3 (48) | 9.4±1.5 |
| B | 22.7382,120.2583 |  | 6.3 (48) | 8.3 (48) | 7.3±1.5 |
| C | 22.7353,120.2813 |  | 10.4 (48) | 12.5 (48) | 11.4±1.5 |
| D | 22.7073,120.2853 |  | 29.2 (48) | 29.2 (48) | 29.2±0* |
| E | 22.7190,120.2993 |  | 33.3 (48) | 29.2 (48) | 31.3±2.9* |
| F | 22.6811,120.2830 |  | 31.3 (48) | 39.6 (48) | 35.4±5.9* |
| G | 22.7017,120.3020 |  | 29.0 (254) | 52.4 (254) | 40.7±16.6* |
| H | 22.6725,120.3014 |  | 6.3 (48) | 12.5 (48) | 9.3±4.4 |
| I | 22.6922,120.3249 |  | 7.3 (254) | 11.6 (254) | 9.4±3.0 |
| J | 22.7554,120.3321 |  | 10.4 (48) | 8.3 (48) | 9.4±1.5 |
| K | 22.7377,120.3391 |  | 8.3 (48) | 6.3 (48) | 7.3±1.5 |
| L | 22.7218,120.3618 |  | 10.4 (48) | 8.3 (48) | 9.4±1.5 |

*: Significant difference (t-test, p<0.05) compared with site A-C and site H-L
