# Supplementary material for: Airborne Transmission of Melioidosis to Humans from Environmental Aerosols Contaminated with B. pseudomallei
Source: PLoS Negl Trop Dis. 2015 Jun 10;9(6):e0003834. doi: 10.1371/journal.pntd.0003834 (PMC4462588; doi:10.1371/journal.pntd.0003834)
Supplement: S4 Table — (DOCX) [file pntd.0003834.s006.docx]

**S4 Table. Strain information, ST types and alleles of *B. pseudomallei* isolates**

|  |  |  |  | Housekeeping genes | | | | | | |
| --- | --- | --- | --- | --- | --- | --- | --- | --- | --- | --- |
| Strain | Source | Year | ST type | ace | gltB | gmhD | lepA | lipA | narK | ndh |
| NKS01 | Aerosols | 2013 | 58 | 3 | 1 | 5 | 1 | 1 | 4 | 1 |
| NKS02 | Aerosols | 2013 | 58 | 3 | 1 | 5 | 1 | 1 | 4 | 1 |
| NKS03 | Aerosols | 2013 | 58 | 3 | 1 | 5 | 1 | 1 | 4 | 1 |
| NKS04 | Surficial soil | 2012 | 58 | 3 | 1 | 5 | 1 | 1 | 4 | 1 |
| NKS06 | Surficial soil | 2012 | 58 | 3 | 1 | 5 | 1 | 1 | 4 | 1 |
| NKS07 | Shallow soil | 2012 | 58 | 3 | 1 | 5 | 1 | 1 | 4 | 1 |
| NKS08 | Deep soil | 2012 | 58 | 3 | 1 | 5 | 1 | 1 | 4 | 1 |
| NKS13 | Deep soil | 2013 | 1354 | 4 | 154 | 5 | 113 | 1 | 29 | 1 |
| NKS15 | Deep soil | 2013 | 58 | 3 | 1 | 5 | 1 | 1 | 4 | 1 |
| NKS16 | Deep soil | 2012 | 834 | 3 | 1 | 6 | 1 | 1 | 29 | 1 |
| NKS18 | Deep soil | 2013 | 704 | 1 | 1 | 6 | 1 | 1 | 29 | 1 |
| NKS19 | Deep soil | 2012 | 1001 | 4 | 2 | 6 | 1 | 1 | 29 | 1 |
| NKS20 | Deep soil | 2013 | 58 | 3 | 1 | 5 | 1 | 1 | 4 | 1 |
| NKS26 | Shallow soil | 2012 | 1115 | 1 | 2 | 6 | 1 | 1 | 29 | 1 |
| NKS27 | Shallow soil | 2012 | 1001 | 4 | 2 | 6 | 1 | 1 | 29 | 1 |
| NKS28 | Shallow soil | 2012 | 1001 | 4 | 2 | 6 | 1 | 1 | 29 | 1 |
| NKS29 | Surficial soil | 2012 | 1001 | 4 | 2 | 6 | 1 | 1 | 29 | 1 |
| NKS30 | Aerosols | 2013 | 58 | 3 | 1 | 5 | 1 | 1 | 4 | 1 |
| NKS32 | Surficial soil | 2012 | 834 | 3 | 1 | 6 | 1 | 1 | 29 | 1 |
| NKS33 | Surficial soil | 2013 | 58 | 3 | 1 | 5 | 1 | 1 | 4 | 1 |
| NKS34 | Surficial soil | 2013 | 58 | 3 | 1 | 5 | 1 | 1 | 4 | 1 |
| NKS35 | Surficial soil | 2013 | 58 | 3 | 1 | 5 | 1 | 1 | 4 | 1 |
| NKS37 | Shallow soil | 2013 | 1001 | 4 | 2 | 6 | 1 | 1 | 29 | 1 |
| NKS38 | Shallow soil | 2013 | 58 | 3 | 1 | 5 | 1 | 1 | 4 | 1 |
| NKS39 | Shallow soil | 2013 | 58 | 3 | 1 | 5 | 1 | 1 | 4 | 1 |
| NKS40 | Surficial soil | 2013 | 1001 | 4 | 2 | 6 | 1 | 1 | 29 | 1 |
| BPS1004 | Human | 2010 | 58 | 3 | 1 | 5 | 1 | 1 | 4 | 1 |
| BPS1027 | Human | 2010 | 58 | 3 | 1 | 5 | 1 | 1 | 4 | 1 |
| BPS1028 | Human | 2010 | 58 | 3 | 1 | 5 | 1 | 1 | 4 | 1 |
| BPS1031 | Human | 2010 | 58 | 3 | 1 | 5 | 1 | 1 | 4 | 1 |
| BPS1033 | Human | 2010 | 58 | 3 | 1 | 5 | 1 | 1 | 4 | 1 |
| BPS1034 | Human | 2010 | 58 | 3 | 1 | 5 | 1 | 1 | 4 | 1 |
| BPS1035 | Human | 2010 | 58 | 3 | 1 | 5 | 1 | 1 | 4 | 1 |
| BPS1044 | Human | 2010 | 58 | 3 | 1 | 5 | 1 | 1 | 4 | 1 |
| BPS1047 | Human | 2010 | 58 | 3 | 1 | 5 | 1 | 1 | 4 | 1 |
| BPS1112 | Human | 2011 | 58 | 3 | 1 | 5 | 1 | 1 | 4 | 1 |
| BPS1115 | Human | 2011 | 58 | 3 | 1 | 5 | 1 | 1 | 4 | 1 |
| BPS1136 | Human | 2011 | 58 | 3 | 1 | 5 | 1 | 1 | 4 | 1 |
| BPS1138 | Human | 2011 | 58 | 3 | 1 | 5 | 1 | 1 | 4 | 1 |
| BPS1144 | Human | 2011 | 58 | 3 | 1 | 5 | 1 | 1 | 4 | 1 |
| BPS1203 | Human | 2012 | 58 | 3 | 1 | 5 | 1 | 1 | 4 | 1 |
| BPS1208 | Human | 2012 | 58 | 3 | 1 | 5 | 1 | 1 | 4 | 1 |
| BPS1219 | Human | 2012 | 58 | 3 | 1 | 5 | 1 | 1 | 4 | 1 |
| BPS1221 | Human | 2012 | 58 | 3 | 1 | 5 | 1 | 1 | 4 | 1 |
| BPS1224 | Human | 2012 | 58 | 3 | 1 | 5 | 1 | 1 | 4 | 1 |
| BPS1304 | Human | 2013 | 58 | 3 | 1 | 5 | 1 | 1 | 4 | 1 |
| BPS1306 | Human | 2013 | 58 | 3 | 1 | 5 | 1 | 1 | 4 | 1 |
